# 2517. Activity of Sulbactam-durlobactam, Antibacterial Combinations, and Comparators Against a Challenge Set of 66 *Acinetobacter baumannii-calcoaceticus* Species Complex Isolates

**DOI:** 10.1093/ofid/ofad500.2135

**Published:** 2023-11-27

**Authors:** Michael D Huband, Rodrigo E Mendes, Gina M Morgan, Holly Huynh, Mariana Castanheira

**Affiliations:** JMI Laboratories, North Liberty, Iowa; JMI Laboratories, North Liberty, Iowa; JMI Laboratories, North Liberty, Iowa; JMI Laboratories, North Liberty, Iowa; JMI Laboratories, North Liberty, Iowa

## Abstract

**Background:**

Sulbactam-durlobactam (SUL-DUR) is in clinical development for the treatment of *Acinetobacter baumannii calcoaceticus* species complex (ACB) isolates, including multidrug-resistant (MDR) and carbapenem-resistant strains. SUL-DUR is a combination of sulbactam, a β-lactam antibacterial with activity against ACB and durlobactam, a β-lactamase inhibitor with activity against Class A, C and D β-lactamases. In this study, we evaluated the *in vitro* activity of SUL-DUR, as well as double and triple combinations of SUL, DUR and cefiderocol or imipenem against a set of 66 ACB isolates.

Activity of sulbactam-durlobactam (fixed 4 mg/L), antibacterial combinations, and comparators against a set of 66 A. baumannii-calcoaceticus species complex isolates

**Figure:**
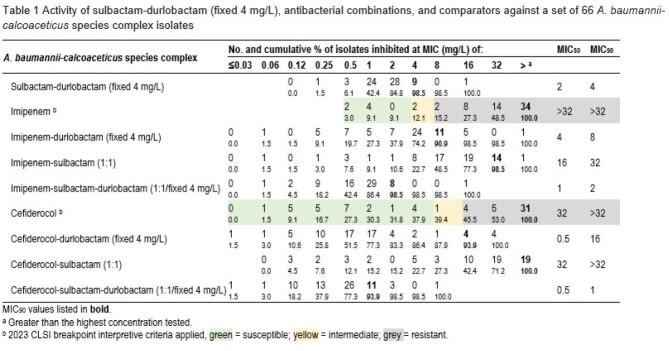

**Methods:**

Bacterial isolates consisted of 41 ACB isolates from the Centers for Disease Control and Prevention Antimicrobial Resistance Bank and 25 molecularly characterized cefiderocol-resistant ACB clinical isolates from the SENTRY Antimicrobial Surveillance Program. ACB identifications were confirmed by MALDI-TOF. Susceptibility testing of SUL-DUR and comparators was conducted according to CLSI M07 (2018) and M100 (2023) guidelines. Susceptibility testing of cefiderocol and cefiderocol combinations was conducted in Chelex-treated Mueller-Hinton broth. Cefiderocol and imipenem results were interpreted using CLSI breakpoint criteria

**Results:**

SUL-DUR (MIC_50/90_, 2/4 mg/L; 98.5% inhibited at ≤4 mg/L), cefiderocol-sulbactam-durlobactam (MIC_50/90_, 0.5/1 mg/L; 98.5% inhibited at ≤4 mg/L), and imipenem-sulbactam-durlobactam (MIC_90_, 1/2 mg/L; 98.5% inhibited at ≤2 mg/L) were the most active combinations tested against the set of 66 *A. baumannii* isolates (Table). Against these isolates, cefiderocol and imipenem susceptibilities were 37.9% (CLSI) and 9.1% (CLSI), respectively.

**Conclusion:**

Overall, SUL-DUR (MIC_50/90_, 2/4 mg/L; 98.5% inhibited at ≤4 mg/L) was active against the ACB isolates tested regardless of cefiderocol susceptibility, including MDR and carbapenem-resistant isolates. The addition of imipenem or cefiderocol to SUL-DUR did not greatly improve SUL-DUR activity (decreased overall MIC_90_ values by up to 4-fold) when compared to SUL-DUR tested alone. The potent activity of SUL-DUR against this set of ACB isolates, including cefiderocol-resistant strains, supports the continued development of this combination.

**Disclosures:**

**Michael D. Huband, BS**, BARDA: This study has been funded in part by BARDA under Contract No. 75A50120C00001.|Entasis: Grant/Research Support|Paratek: Grant/Research Support|Pfizer: Grant/Research Support **Rodrigo E. Mendes, PhD**, AbbVie: Grant/Research Support|Basilea: Grant/Research Support|Cipla: Grant/Research Support|Entasis: Grant/Research Support|GSK: Grant/Research Support|Paratek: Grant/Research Support|Pfizer: Grant/Research Support|Shionogi: Grant/Research Support **Gina M. Morgan, MS**, Entasis: Grant/Research Support **Holly Huynh, BS**, Entasis: Grant/Research Support **Mariana Castanheira, PhD**, AbbVie: Grant/Research Support|Basilea: Grant/Research Support|bioMerieux: Grant/Research Support|Cipla: Grant/Research Support|CorMedix: Grant/Research Support|Entasis: Grant/Research Support|Melinta: Grant/Research Support|Paratek: Grant/Research Support|Pfizer: Grant/Research Support|Shionogi: Grant/Research Support

